# Differential Mobilization of the Phospholipid and Triacylglycerol Pools of Arachidonic Acid in Murine Macrophages

**DOI:** 10.3390/biom12121851

**Published:** 2022-12-11

**Authors:** Miguel A. Bermúdez, Julio M. Rubio, María A. Balboa, Jesús Balsinde

**Affiliations:** 1 Instituto de Biología y Genética Molecular, Consejo Superior de Investigaciones Científicas (CSIC), 47003 Valladolid, Spain; 2 Centro de Investigación Biomédica en Red de Diabetes y Enfermedades Metabólicas Asociadas (CIBERDEM), Instituto de Salud Carlos III, 28029 Madrid, Spain

**Keywords:** arachidonic acid, membrane phospholipid, triacylglycerol, phospholipase A_2_, inflammation, monocytes/macrophages

## Abstract

Innate immune cells such as monocytes and macrophages contain high levels of arachidonic acid (AA), part of which can be mobilized during cellular activation for the formation of a vast array of bioactive oxygenated metabolites. Monocytes and macrophages present in inflammatory foci typically incorporate large amounts of AA, not only in membrane phospholipids, but also in neutral lipids such as triacylglycerol. Thus, it was of interest to investigate the metabolic fate of these two AA pools in macrophages. Utilizing a variety of radiolabeling techniques to distinguish the phospholipid and triacylglycerol pools, we show in this paper that during an acute stimulation of the macrophages with yeast-derived zymosan, the membrane phospholipid AA pool acts as the major, if not the only, source of releasable AA. On the contrary, the AA pool in triacylglycerol appears to be used at a later stage, when the zymosan-stimulated response has declined, as a source to replenish the phospholipid pools that were consumed during the activation process. Thus, phospholipids and triacylglycerol play different in roles AA metabolism and dynamics during macrophage activation.

## 1. Introduction

Arachidonic acid (AA, 20:4n−6) is the most abundant polyunsaturated (PUFA) fatty acid in major immunoinflammatory cells [1]. It may be synthesized from its essential precursor, linoleic acid (18:2n−6), or obtained directly as such from the diet [2]. In innate immune cells, AA is incorporated primarily into the sn-2 position of membrane glycerophospholipids via a highly-regulated pathway of deacylation/reacylation reactions known as the Lands pathway [3,4]. Phospholipids formed de novo, which contain primarily saturated or monounsaturated fatty acids at the sn-2 position, are hydrolyzed by the action of housekeeping phospholipase A_2_s [5,6,7,8] to generate a 2-lysophospholipid (primarily 1-acyl-lysoPC but also 1-acyl-lyso-PI) which is rapidly reacylated with AA to reconstitute a full phospholipid by the combined action of acyl-CoA synthetases and CoA-dependent acyltransferases [9,10,11].

While it is well established that the Lands pathway constitutes the prime pathway for phospholipid fatty acid recycling and the entry of AA into cell phospholipids [12], it is also important to consider that further fatty acid remodeling reactions take place, through which the AA is moved from 1-ester-linked (primarily choline-containing phospholipids, PC) to 1-ether-linked (1-alkyl and 1 alk-1-enyl) phospholipids (primarily ethanolamine-containing phospholipids, PE) via CoA-independent transacylation reactions [13,14,15,16,17]. This highly integrated set of lipid metabolic routes has revealed the diversity of functions that the various cellular phospholipid classes may serve with regard to the utilization of AA and likely other PUFA such as those of the n−3 series, eicosapentaenoic acid (EPA; 20:5n−3) and docosahexaenoic acid (DHA; 22:6n−3). Thus, some phospholipids act as initial acceptors of the AA (mostly diacyl-PC and PI), whereas others constitute major stable cellular reservoirs of esterified AA (e.g., alk-1-enyl-PE, also known as ethanolamine plasmalogens) [13,14,15,16,17]. Overall, phospholipid fatty acid remodeling reactions involving AA results in the cells showcasing an ample variety of AA-containing phospholipid species, which may play important roles for the execution of certain biological responses [18,19,20,21]. In particular, achieving an appropriate distribution of AA between cellular phospholipid pools is key for the biosynthesis of AA-derived eicosanoids. This is so because the nature and amount of oxylipins produced under activation conditions may ultimately depend on the composition of the subcellular site where the relevant AA-hydrolyzing phospholipase A_2_ acts [22,23,24,25,26,27].

The mechanisms responsible for the intracellular movement of AA between the different phospholipid classes and subclasses have been extensively characterized at the molecular species level, even though the molecular identity of the key enzyme involved, CoA-dependent transacylase, is yet to be determined [13,14,15,16,17,28,29,30]. However, the trafficking of AA between phospholipids and neutral lipids, particularly triacylglycerol (TAG), has received much less attention. This is probably due to the finding that most innate immune cell populations generally contain low amounts of AA in neutral lipids in the resting state [31,32]. However, under pathophysiological situations such as lung infection and inflammation, or endothelial cell damage leading to atheroma plaque buildup, phagocytic cells capture large amounts of AA and incorporate it into the triacylglycerol (TAG) fraction within cytoplasmic lipid droplets [33,34,35]. Whether the AA stored in neutral lipids constitutes a significant source of free fatty acid for the synthesis of lipid mediators is unclear, and may depend on cell type and activation conditions [33,34,35,36,37,38]. However, other possible pathophysiological implications for the presence of AA in TAG have not been thoroughly investigated. It has been suggested that AA-containing TAG may represent an expandable pool that is used by the cells when the glycerophospholipid membrane pool is replete. In this manner, AA entry into TAG would protect the cells from the deleterious effects produced by high concentrations of the free fatty acid and, in turn, help regulate the level of free AA available for lipid mediator synthesis [39,40]. An intriguing possibility that follows from these observations is that the TAG pool could represent a repository of AA for eventual transfer to membrane phospholipids. This would serve to replenish the latter pool, which is partially depleted as a consequence of cellular activation. In this work, we have investigated the possible existence of this AA transfer pathway between TAG and phospholipids in murine peritoneal macrophages. These cells contain high amounts of AA esterified in membrane glycerophospholipids, which is readily mobilized in response to receptor stimulation [41,42,43,44,45,46,47,48,49,50,51]. The experiments shown in this study reveal that AA stored in TAG indeed constitutes a meaningful pool of fatty acid destined to replenish the phospholipid AA pool. Thus, TAG and phospholipid pools serve different roles in the regulation of AA metabolism during macrophage activation.

## 2. Materials and Methods

### 2.1. Reagents

Cell culture medium was from Molecular Probes-Invitrogen (Carlsbad, CA, USA). Organic solvents (Optima^®^ LC/MS grade) were from Fisher Scientific (Madrid, Spain). Lipid standards were from Avanti (Alabaster, AL, USA) or Cayman (Ann Arbor, MI, USA). Silicagel G thin-layer chromatography plates were from Macherey-Nagel (Düren, Germany). [5,6,8,9,11,12,14,15-^3^H]Arachidonic acid (180 Ci/mmol) and [1-^14^C]arachidonic acid (50 µCi/mmol) were from PerkinElmer (Boston, MA, USA). Inhibitors were from Cayman. All other reagents were from Sigma-Aldrich (Madrid, Spain).

### 2.2. Cell Culture and Stimulation Conditions

Resident peritoneal macrophages from Swiss male mice (University of Valladolid Animal House, 10–12 weeks old) were obtained by peritoneal lavage using 5 mL cold phosphate-buffered saline, and purified by adherence to 35-mm plastic culture dishes (Costar, Cambridge, MA, USA) as described elsewhere [51]. The cells were cultured in RPMI 1640 (1.5 × l0^6^ cells/mL; 2-mL final volume) with 10% heat-inactivated calf serum, 100 U/mL penicillin, and 100 μg/mL streptomycin. All procedures involving animals were undertaken under the supervision of the Institutional Committee of Animal Care and Usage of the University of Valladolid (Approval No. 7406000), and are in accordance with the guidelines established by the Spanish Ministry of Agriculture, Food, and Environment and the European Union.

The cells were placed in serum-free medium for 1 h. Afterward, they were challenged by the stimuli for the times indicated. When inhibitors were used, they were added 15-30 min before the addition of stimuli. The inhibitors were dissolved in dimethyl sulfoxide. The appropriate controls were included to ensure that the dimethyl sulfoxide had no effect on any of the responses measured. Zymosan was prepared as described [52]. Only zymosan batches that demonstrated no endogenous phospholipase A_2_ activity, as measured by in vitro assay [53,54,55], were used in this study. Cell protein content was quantified using a commercial kit (BioRad Protein Assay, Bio-Rad, Hercules, CA, USA) [56]. 

Radiolabeling of the cells with [^3^H]AA was achieved by including 0.25 µCi/mL [^3^H]AA at either 5 nM or 20 µM during the overnight adherence period (20 h). The AA was dissolved in ethanol. It was added to the cells after dilution with fresh medium. Appropriate controls were run in parallel to exclude an effect of the solvent on cells. AA that had not been incorporated into cellular lipids was removed by washing the cells four times with serum-free medium containing 0.5 mg/mL albumin. These [^3^H]AA-labeled cells were used for AA release experiments, and the incubations were performed in the presence of 0.5 mg/mL bovine serum albumin (fatty acid-free) [57,58,59]. After incubation with the stimuli, the supernatants were removed, cleared of detached cells by centrifugation and assayed for radioactivity by liquid scintillation counting. For labeling with [^3^H]AA and [^14^C]AA, the cells were incubated with 0.25 µCi/mL [^3^H]AA for 20 h and then with 0.1 µCi/mL [^14^C]AA for 2 h. Non-incorporated AA was removed by washing the cells four times with serum-free medium containing 0.5 mg/mL albumin. After the stimulations, the supernatants were assayed for radioactivity. The cell monolayers were homogenized and the lipids were extracted according to Bligh and Dyer [60]. Phospholipids were separated from neutral lipids by thin-layer chromatography, using *n*-hexane/diethyl ether/acetic acid (70:30:1, v/v/v) [61]. The bands corresponding to the different lipid classes were scraped from the plates and their radioactive content was determined by scintillation counting using a Beckman Coulter LS6500 Liquid Scintillation Counter (Beckman, Fullerton, CA, USA). 

### 2.3. Gas Chromatography/Mass Spectrometry (GC-MS) Analyses

Phospholipids and TAG were separated by thin-layer chromatography. The solvent system used was *n*-hexane/diethyl ether/acetic acid (70:30:1, v/v/v) [61]. Areas containing the lipids of interest were scraped from the plate, and subjected to transmethylation with 0.5 M KOH in methanol for 60 min at 37 °C [62,63,64,65]. 

GC-MS analyses of fatty acid methyl esters were performed using an Agilent 7890A gas chromatograph coupled to an Agilent 5975C mass-selective detector operated in electron impact mode, equipped with an Agilent 7693 autosampler. An Agilent DB23 column was used (60 m length × 0.25 mm internal diameter × 0.15 µm film thickness) (Agilent Technologies, Santa Clara, CA, USA) was used.

### 2.4. Data Analysis

The results are shown as means ± standard error of the mean and were analyzed for statistical significance by *t*-test (two groups) or by ANOVA (more than two groups), followed by Tukey’s post hoc test, using GraphPad Prism software. A value of *p* < 0.05 was considered statistically significant.

## 3. Results

**Figure 1:**
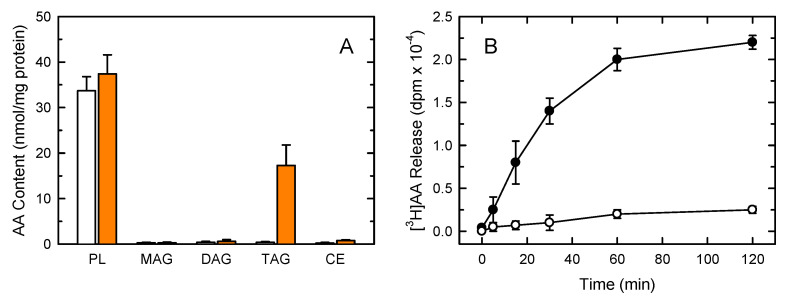
AA distribution in murine macrophages. (**A**) AA incorporation into the lipids of mouse peritoneal macrophages. The cells were either untreated (open bars) or treated with 20 µM AA (orange bars). Afterward, the various lipid classes were isolated and their AA content was measured by GC-MS. (**B**) The cells, prelabeled with [^3^H]AA, were either untreated (open symbols) or treated with 0.5 mg/mL zymosan (black symbols) for the times indicated. Afterward, the extracellular media were removed and analyzed for ^3^H-radioactivity content. The data are expressed as mean values ± S.E.M. (*n* = 6).

Resident peritoneal macrophages contain relatively large amounts of AA esterified in phospholipids, and rather low amounts in neutral lipids (Figure 1A). However, macrophages in inflammatory foci, which are exposed to high quantities of exogenous free AA arising from the inflammatory milieu [40,66], can efficiently take up the fatty acid and incorporate it into TAG. Thus, under pathophysiologically relevant conditions the macrophages contain significant amounts of AA in TAG, which could be mobilized to serve specific functions [67,68,69]. It has been shown that these AA-laden macrophages can be obtained in vitro by incubating the cell cultures with exogenous AA [40], which provides a direct means to examine the regulatory features and significance of AA accumulation in neutral lipids. In accordance with the above, Figure 1A shows that incubating the macrophages with 20 µM AA for 20 h greatly increased the AA content in TAG. Importantly, the phospholipid (PL) fraction increased only slightly its AA content, suggesting that this class is already full in the resting state and cannot accommodate more fatty acid. 

AA incorporation into cholesterol esters (CE) was very low. This finding, which has also been observed in human monocytes [70], may suggest that the acyltransferase using AA-CoA as a donor shows little affinity for free cholesterol as an acceptor. Cell viability was not affected by the AA loading. This was an important measurement to conduct, since defects in AA incorporation into cellular lipids may lead to apoptotic cell death [71,72]. 

After labeling the cellular AA pools with radioactive fatty acid (incubation with 20 µM [^3^H]AA for 20 h), the time course of AA release induced by yeast-derived zymosan was determined in the AA-laden cells (Figure 1B). We chose zymosan for our studies because this stimulus has been used for years as a model to analyze the intracellular signaling pathways for lipid mediator production in murine macrophages [24,41,43,44,45,46,73,74,75,76,77,78]. Consistent with previous reports [76,77,78], AA release proceeded linearly up to 60 min, then reaching a plateau at 120 min.

**Figure 2:**
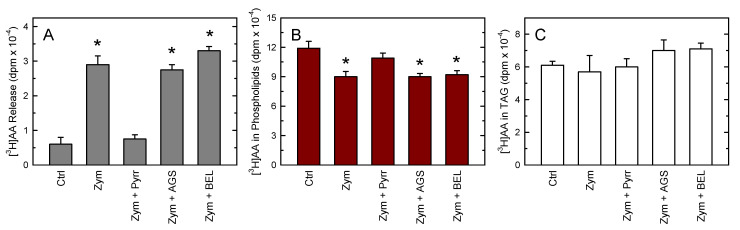
AA mobilization by zymosan-stimulated peritoneal macrophages. The [^3^H]AA-labeled cells were either untreated (Ctrl) or stimulated with 0.5 mg/mL zymosan for 2 h in the absence (Zym) or presence of 1 µM pyrrophenone (Zym + Pyrr), or 20 µM atglistatin (Zym + AGS), or 10 µM bromoenol lactone (Zym + BEL). Afterward, the [^3^H]AA released to the supernatants (**A**) or remaining in phospholipids (**B**) or TAG (**C**) was quantified as described in Materials and Methods. The data are expressed as mean values ± S.E.M. (*n* = 6). * *p* < 0.05, significantly different from untreated cells.

To assess the contribution of PL and TAG pools to overall AA release, we first analyzed the AA content in these fractions after stimulating the cells with zymosan (Figure 2). At the time the cells released abundant [^3^H]AA to the extracellular medium (Figure 2A), significant decreases of AA in PL were seen as well (Figure 2B). Note that, in accordance with previous reports [75,78], most of the fatty acid remained bound to PL after the activation process; thus, the AA released to the extracellular medium represented only a small fraction of total cellular AA. Conversely, no significant AA decreases in the TAG fraction could be detected (Figure 2C). These data suggest that the bulk of AA released after the zymosan stimulations arises from PL, not from TAG.

To further substantiate the above findings, selective inhibitors of cytosolic phospholipase A_2_α (cPLA_2_α) and TAG lipase were used next to block the AA release from phospholipids and TAG, respectively. Chemical inhibitors target functions that depend on enzyme activity, sparing non-catalytic functions, and induce no compensatory mechanisms at the enzyme expression level. In addition, enzyme inhibition develops rapidly, which reduces the impact of long-term nonspecific effects. To inhibit cPLA_2_α, we used the well-established inhibitor pyrrophenone [79,80,81]. To block TAG hydrolysis, we used atglistatin, a potent and selective inhibitor of murine TAG lipase [38,82]. For comparative purposes with data in the bibliography [35,83,84], we also used bromoenol lactone (BEL), a general inhibitor of patatin-like phospholipases that has been shown to efficiently prevent TAG lipase-induced TAG hydrolysis [85,86,87]. As shown in Figure 2, pyrrophenone blocked zymosan-induced AA release to almost basal levels, and also strongly prevented the AA loss from PL, while having no effect on TAG levels. Figure 2 also shows that neither atglistatin nor BEL exerted significant effects on zymosan-stimulated AA release or affected AA levels in phospholipids or TAG.

**Figure 3:**
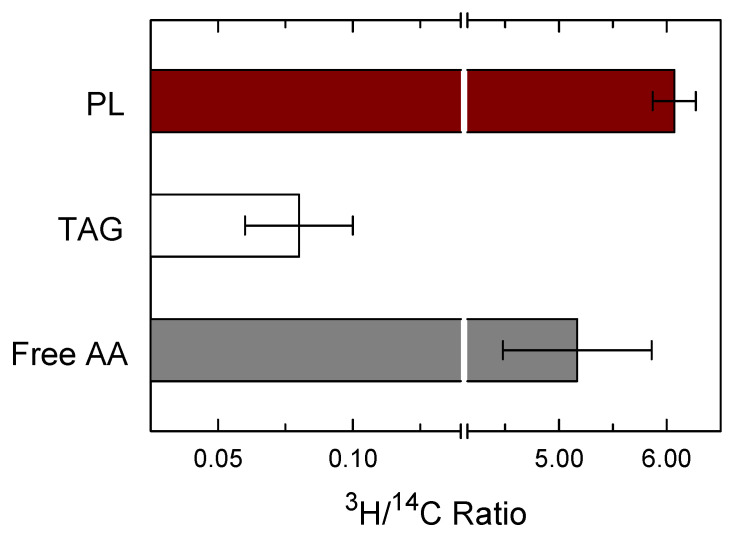
Source of AA release in zymosan-stimulated macrophages. The cells, labeled with [^3^H]AA and [^14^C]AA were treated with 0.5 mg/mL zymosan for 1 h. Afterward, the ^3^H/^14^C ratios of extracellularly liberated free AA, PL and TAG were calculated. The data are expressed as mean values ± S.E.M. (*n* = 6).

While the results presented in Figure 2 provide strong evidence that PL, not TAG, constitutes the main source for the AA release from zymosan-stimulated macrophages, the experimental conditions utilized for labeling the cells with radioactive AA do not permit to differentiate between the fatty acid present at either lipid class. To accomplish this, we took advantage of the fact that the cellular AA pools of PL and TAG can be differentiated by double-labeling them with [^3^H]AA and [^14^C]AA at different concentrations [33]. Thus, low concentrations of [^3^H]AA were used to label the PL and high concentrations of [^14^C]AA to label mainly the TAG. The cells were first labeled with 5 nM [^3^H]AA for 20 h, a time frame long enough to allow for the radiolabeled fatty acid to equilibrate among lipid classes and thus resemble the endogenous distribution of AA [88,89]. Under these conditions, most of the ^3^H-radioactivity (93.0 ± 0.6%) was associated with the PL fraction, with very low amounts being found in the TAG fraction (7.0 ± 0.6%). After the 20-h incubation period with [^3^H]AA, the cells were washed and pulse-labeled with 20 µM [^14^C]AA for 2 h. At these high concentrations and consistent with the results of Figure 1, a substantial part of the ^14^C-radioactivity is incorporated into TAG, with PL incorporating lower amounts [33]. This disparate labeling of the cells with [^3^H]AA and [^14^C]AA produces two very different ^3^H/^14^C ratios for PL versus TAG (Figure 3), making this strategy very effective to discriminate between the two cellular AA pools [33]. Subsequent to the double-labeling, the cells were treated with 0.5 mg/mL zymosan for 1 h, and the ^3^H/^14^C ratio was determined for the free AA released to the incubation medium. The ^3^H/^14^C ratios in PL and TAG did not change with stimulation. As shown in Figure 3, after stimulation, the ^3^H/^14^C ratio of the released free AA (5.17 ± 0.69) was found to be much closer to the PL ratio (6.07 ± 0.20) than to the TAG ratio (0.08 ± 0.01). Thus, these data further support the notion that the bulk of released AA arises primarily from PL, not from TAG.

**Figure 4:**
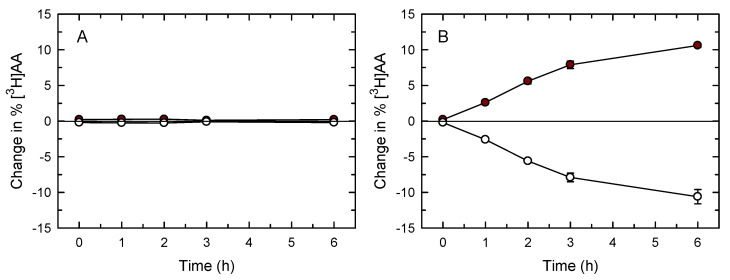
Transfer of [^3^H]AA from TAG to phospholipids. Cells prelabeled with [^3^H]AA were either untreated (**A**) or stimulated with 0.5 mg/mL zymosan for 2 h (**B**). Afterward, they were washed and transferred to fresh media, and the distribution of [^3^H]AA content in phospholipids (maroon symbols) and TAG (open symbols) was determined at the times indicated. The data are expressed as mean values ± S.E.M. (*n* = 6).

To identify other possible metabolic fates for the AA present in TAG, we analyzed whether the mixing of AA pools (i.e., transfer of AA moieties from TAG to PL or *vice versa*) occurred in the macrophages along the course of our experiments. To this end, the cells were labeled first with 5 nM [^3^H]AA and then with 20 µM [^3^H]AA for 20 h. This results in substantial ^3^H-labeling of both PL and TAG pools (66.2 ± 4.0% of the total cellular label being present in PL, and 33.8 ± 1.4% in TAG; mean ± SEM, *n* = 6). Afterward, the cells were stimulated or not stimulated with zymosan for 2 h, washed, and incubated in fresh media without stimulus. At different time periods, the amount of radioactivity in PL and TAG was estimated. Note that, as depicted in Figure 1B, the zymosan-stimulated AA release response has ceased or substantially declined after a 2-h incubation. The data in Figure 4 show that a significant movement of [^3^H]AA from TAG to PL was detected in the zymosan-stimulated cells, but not in the otherwise untreated cells. These data indicate that, while in resting macrophages the AA pools in TAG and PL remain stable, there is a transfer of AA from the TAG pool to the phospholipid pool in the cells that had previously been stimulated. 

## 4. Discussion

Innate immune cells such as neutrophils, monocytes and macrophages are cells particularly enriched in AA, and thus are able to generate high amounts of eicosanoids under immunoinflammatory conditions [90]. Two major routes have been described for the incorporation of AA into the lipids of these cells [9,13]. The first one operates at low, physiological levels of free AA, and results in the fatty acid being incorporated into phospholipids via the Lands pathway of fatty acid recycling. The second route operates when the cells are exposed to high concentrations of free AA, which occurs primarily under pathophysiological conditions (i.e., in inflammatory foci), and leads to the accumulation of large amounts of the fatty acid in TAG [9,13]. This route of AA entry into TAG represents a high capacity, low affinity pathway that is turned on after the higher affinity Lands cycle becomes overflown because of the high availability of free AA [9,13]. Our current results showing that incubation of the macrophages with exogenous AA results in the accumulation of the fatty acid into TAG with little change in phospholipid AA levels are in fully agreement with this view. 

In accordance with these observations, circulating blood neutrophils and monocytes, and resident macrophages, contain very low amounts of AA in TAG under physiological conditions [31,32,35,91]. Thus, the acute eicosanoid response of these cells to innate stimuli depends on the mobilization of AA from membrane phospholipid pools. The high capacity of these cells to incorporate very large amounts of AA into TAG under pathophysiological circumstances raises the question of its significance. AA entry into TAG may represent a means for the cells to store excess fatty acid and, in this manner, protect themselves from possible toxic effects of the free fatty at high concentrations. However, other metabolic fates are possible. An obvious possibility is that the accumulation of AA into TAG allows the cells to use an alternate pathway for the mobilization of free AA for conversion into bioactive oxylipins [67,68]. We have addressed this question in this study with murine peritoneal macrophages. Our results utilizing a variety of radiolabeling strategies to differentiate the cellular AA pools in TAG and PL strongly suggest that AA liberated in response to cellular stimulation arises mostly from PL, and the involvement of TAG in this process, if there is any, is minor. Our results in this regard are consistent with previous works in human monocytes and macrophages [33,34,35,89], which also failed to detect a significant involvement of TAG in stimulus-induced AA release. While differences regarding the intracellular sites of action of secreted phospholipase A_2_ [92,93], but not of cPLA_2_α [94,95], have been found between human and murine macrophages, the AA mobilization responses of human and murine macrophages are, in general, comparable. Both human and murine macrophages produce high quantities of prostaglandin E_2_ from the cyclooxygenase pathway, and 5-HETE from the lipoxygenase pathway [96,97].

Importantly, at late time points (>180 min), after the zymosan-stimulated AA release has ceased, we detected a slow but sustained decrease in AA levels in TAG and a concomitant increase in AA in phospholipids. This was not observed in the unstimulated cells, suggesting that the AA pools in the resting state are stable and do not interact with each other. We assume that this is probably a reflection of the finding that the phospholipid AA pool in the resident macrophages is already replete. 

The identification of a continued transfer of AA moieties from TAG to phospholipids after the stimulated AA release response has ended or nearing the end may constitute an important mechanism for the cell to return to homeostasis. By repleting the early releasable AA pools, i.e., those residing in membrane phospholipids, the macrophage would be capable of responding again to innate stimuli with a full AA release response. While such a mechanism applies to stimuli that promote the immediate mobilization of AA via rapid receptor-activation of cPLA_2_α, it is important to consider as well that there are also stimuli that promote delayed AA mobilization responses (i.e., spanning several hours) [98,99]. Thus, it is possible that, under these circumstances, the AA present in TAG may contribute significantly to the overall AA mobilization response by acting as a late releasing AA pool. The final outcome would be a long-term recovery of AA levels in phospholipids at the expense of net fatty acid losses from TAG. A scenario such as this could be particularly relevant to mast cells, which experience AA mobilization responses to a variety of receptor-directed stimuli [28,99,100]. Depending on anatomical localization, mast cells may contain significant amounts of AA in TAG constitutively [89]. In keeping with these observations, a recent study using human mast cells showed that silencing of TAG lipase diminished eicosanoid production by these cells at least as effectively as silencing cPLA_2_α [36]. Thus, the suggestion was made that, in addition to or independently of being used to replenish emptied phospholipid AA pools, the free AA arising from TAG could be directly used as a substrate for the formation of oxylipins. It should be noted in this regard that studies using mast cells from *Pla2g4a-/-* mice have shown that cPLA_2_α is essential for eicosanoid production [99,100,101]. Aside from species differences, these findings raise the intriguing possibility of whether TAG hydrolysis by TAG lipase is required for the proper activation of cPLA_2_α, at least in mast cells. It seems likely that the biochemical pathways and regulatory mechanisms for AA mobilization may differ not only depending on the cell type considered, but also on the nature of the stimulus utilized to trigger the cellular response [67]. 

Likewise, the transfer of fatty acids from phospholipids to TAG occurs in multiple cell types not involved in innate immune reactions, such as hepatocytes and epithelial cells [102,103]. It is interesting to note in this regard that, under situations of stress, cells are reported to synthesize TAG in a manner that is dependent upon another cellular phospholipase A_2_, i.e., the group VIA enzyme, also known as Ca^2+^-independent phospholipase A_2_β (iPLA_2_β) [103]. TAG synthesis during stress could constitute a survival strategy that recycles phospholipid-bound fatty acids into energy-generating substrates [103]. While this situation is different from the one described in this paper, it is nonetheless significant that, depending on conditions, different phospholipase A_2_ enzymes may act to produce the free fatty acid that is used for a number of metabolic functions involving TAG, namely the iPLA_2_β enzyme under stress conditions, and the cPLA_2_α under inflammatory conditions.

## 5. Conclusions

The results shown in this study define different biological roles for the AA pools in macrophages. Our data indicate that phospholipid pools likely constitute the major, if not the only, source for releasable AA under acute stimulation conditions. On the other hand, our study identifies a novel metabolic fate of the AA-containing in TAG in macrophages, which is that of replenishing the phospholipid pools that have been exhausted after acute stimulation. Thus, the AA pool in neutral lipids may contribute to regulating AA metabolism and dynamics during innate immune activation of the macrophages.

## Data Availability

Data are contained within the article.
